# Performance of a High-Molecular-Weight AM/AA Copolymer in a CO_2_–Water Polymer Hybrid Fracturing Fluid Under High-Temperature and High-Pressure Conditions

**DOI:** 10.3390/polym18030418

**Published:** 2026-02-05

**Authors:** Tengfei Chen, Shutao Zhou, Tingwei Yao, Meilong Fu, Zhigang Wen, Quanhuai Shen

**Affiliations:** 1 State Key Laboratory of Low Carbon Catalysis and Carbon Dioxide Utilization, Yangtze University, Wuhan 430100, China; chentf_cq@petrochina.com.cn (T.C.); wzg728@sina.com (Z.W.); 18571710267@163.com (Q.S.); 2 Xi’an Changqing Chemical Group Co., Ltd., Xi’an 710021, China; zhoust_cq@petrochina.com.cn (S.Z.); yaotw_cq@petrochina.com.cn (T.Y.); 3 National Engineering Laboratory for Exploration and Development of Low-Permeability Oil & Gas Fields, Xi’an 710018, China

**Keywords:** CO_2_–water polymer hybrid fracturing fluid, polymer-based aqueous phase, AM/AA copolymer, phase stability

## Abstract

To reduce water consumption and potential formation damage associated with conventional water-based fracturing fluids while improving the proppant-carrying and flow adaptability of CO_2_-based systems without relying on specialized CO_2_ thickeners, a CO_2_–water polymer hybrid fracturing fluid was developed using an AM/AA copolymer (poly(acrylamide-co-acrylic acid), P(AM-co-AA)) as the thickening agent for the aqueous phase. Systematic experimental investigations were conducted under high-temperature and high-pressure conditions. Fluid-loss tests at different CO_2_ volume fractions show that the CO_2_–water polymer hybrid fracturing fluid system achieves a favorable balance between low fluid loss and structural continuity within the range of 30–50% CO_2_, with the most stable fluid-loss behavior observed at 40% CO_2_. Based on this ratio window, static proppant-carrying experiments indicate controllable settling behavior over a temperature range of 20–80 °C, leading to the selection of 60% polymer-based aqueous phase + 40% CO_2_ as the optimal mixing ratio. Rheological results demonstrate pronounced shear-thinning behavior across a wide thermo-pressure range, with viscosity decreasing systematically with increasing shear rate and temperature while maintaining continuous and reproducible flow responses. Pipe-flow tests further reveal that flow resistance decreases monotonically with increasing flow velocity and temperature, indicating stable transport characteristics. Phase visualization observations show that the CO_2_–water polymer hybrid fracturing fluid system exhibits a uniform milky dispersed appearance under moderate temperature or elevated pressure, whereas bubble-dominated structures and spatial phase separation gradually emerge under high-temperature and relatively low-pressure static conditions, highlighting the sensitivity of phase stability to thermo-pressure conditions. True triaxial hydraulic fracturing experiments confirm that the CO_2_–water polymer hybrid fracturing fluid enables stable fracture initiation and sustained propagation under complex stress conditions. Overall, the results demonstrate that the AM/AA copolymer-based aqueous phase can provide effective viscosity support, proppant-carrying capacity, and flow adaptability for CO_2_–water polymer hybrid fracturing fluid over a wide thermo-pressure range, confirming the feasibility of this approach without the use of specialized CO_2_ thickeners.

## 1. Introduction

In recent years, as conventional oil and gas resources have gradually entered a development stage characterized by high costs and limited reserve growth, unconventional resources, including shale gas, tight gas, and coalbed methane, have become an important component of global oil and gas supply and energy structure optimization [1,2]. Unconventional reservoirs are generally characterized by low porosity, ultra-low permeability, and complex fracture systems, making it difficult to achieve economic production relying solely on natural formation energy [3,4]. At present, water-based fracturing fluids are widely used in field operations; however, their inherent limitations have become increasingly prominent. These limitations include high water consumption, heavy flowback burden, and potential damage to water-sensitive reservoirs, particularly irreversible formation damage caused by polymer residues remaining in the pore structure after fracturing operations [5,6].

To mitigate formation damage induced by water-based fracturing fluids and to improve flowback efficiency, non-aqueous or low-water fracturing technologies dominated by carbon dioxide (CO_2_) have attracted increasing attention. CO_2_ exhibits low viscosity, low interfacial tension, high diffusivity, and favorable flowback characteristics, enabling effective stimulation in low-permeability reservoirs. Previous studies have demonstrated that CO_2_ dry fracturing using liquid or supercritical CO_2_ can achieve effective fracture initiation and propagation under certain conditions, resulting in significant production enhancement in unconventional reservoirs [7,8,9,10,11,12,13,14,15,16]. However, pure CO_2_ dry fracturing generally requires specialized surface equipment and suffers from limited proppant transport capability, which restricts its large-scale field application. To address these limitations, CO_2_ quasi-dry fracturing technology was proposed by introducing a small fraction of polymer-based aqueous fluid into the CO_2_ stream, thereby improving proppant transport and operational flexibility [17,18,19,20]. On this basis, recent review studies have systematically summarized the development status, technical advantages, and engineering limitations of CO_2_ fracturing technologies [21]. Nevertheless, with the continuous extension of reservoir development toward deeper and higher-temperature conditions, conventional quasi-dry fracturing systems increasingly exhibit limitations related to fixed gas–liquid ratios and reliance on specialized CO_2_ thickeners, leading to increased costs and reduced operational adaptability.

Under such circumstances, CO_2_–water polymer hybrid fracturing fluids, constructed by increasing the proportion of the aqueous phase and introducing conventional polymer-based water thickeners, have emerged as a potential technical solution with relatively simple operational procedures and lower costs. In recent years, this type of hybrid fracturing fluid has undergone preliminary field application in unconventional reservoirs. However, as the system is still at an early stage of development, its engineering design and field implementation largely rely on experience derived from polymer-thickened water-based fracturing fluids. Existing studies indicate that polymer thickening systems in water-based fracturing fluids have established relatively mature experimental and theoretical foundations in terms of rheological regulation, structural stability, and adaptability under high-temperature, high-pressure, and high-shear conditions [22,23,24,25,26,27,28]. Meanwhile, with respect to flow friction reduction and proppant transport, high-viscosity friction reducers and surfactant–polymer synergistic systems have demonstrated certain engineering advantages under high-temperature environments [29,30,31]. However, in CO_2_–water polymer hybrid fracturing fluids, the introduction of CO_2_ transforms the system from a single-phase water-based fluid into a gas–liquid mixed-phase system. Consequently, the understanding established for conventional water-based fracturing fluids encounters more complex phase behavior and flow characteristics under such conditions. In particular, under high-temperature and high-pressure environments, the functional role, stability, and coupling mechanisms between the polymer-based aqueous phase and CO_2_ in the hybrid system still lack systematic and quantitative experimental investigation, and the specific role played by CO_2_ within this system has not yet been fully clarified.

Based on the above background, an AM/AA copolymer was selected as the thickening agent for the aqueous phase to construct a CO_2_–water polymer hybrid fracturing fluid in this study. Systematic experimental investigations were conducted under high-temperature and high-pressure conditions. The appropriate CO_2_–water mixing ratio range was determined through fluid-loss and static proppant suspension tests. On this basis, the rheological behavior and pipe-flow friction characteristics of the hybrid fracturing fluid were investigated under different temperature and pressure conditions. In addition, phase visualization experiments were performed to analyze the short-term structural stability of the CO_2_–water polymer hybrid fracturing fluid. Furthermore, true triaxial hydraulic fracturing experiments were carried out to obtain injection pressure responses and fracture morphology characteristics, thereby providing experimental evidence for the feasibility and performance adaptability of polymer-based aqueous phases in CO_2_–water hybrid fracturing fluids.

## 2. Experiments

### 2.1. Experimental Materials and Equipment

#### 2.1.1. Experimental Materials

A high-molecular-weight anionic polymer was used as the aqueous-phase component of the CO_2_–water polymer hybrid fracturing fluid. The polymer is a commercially available acrylamide/acrylic acid copolymer (poly(acrylamide-co-acrylic acid), P(AM-co-AA)), denoted as GF-1, which is supplied by Xi’an Changqing Chemical Group Co., Ltd. (Xi’an, China). The polymer is provided in the form of a white powder and readily dissolves in water to form a transparent solution.

**Figure 1:**
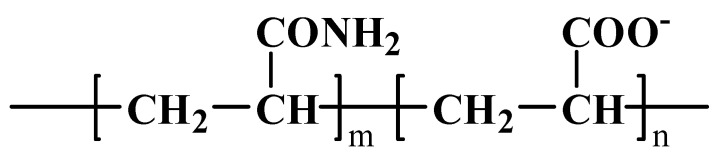
Schematic chemical structure of the AM/AA copolymer.

The molecular weight of the copolymer is on the order of 10^7^ g·mol^−1^. It contains both amide (−CONH_2_) and carboxylate (−COO^−^) functional groups, which contribute to chain flexibility and electrostatic repulsion in aqueous environments. At ambient temperature and pressure, an aqueous solution with a polymer concentration of 0.35 wt% exhibits an apparent viscosity higher than 30 mPa·s. In this study, the polymer was used as received without further purification. The chemical structure of the polymer is schematically illustrated in Figure 1.

Deionized water was used for the preparation of all polymer solutions. Carbon dioxide with a purity of 99.9% was supplied from high-pressure gas cylinders and introduced as the non-aqueous phase in hybrid experiments.

#### 2.1.2. Experimental Equipment

**Figure 2:**
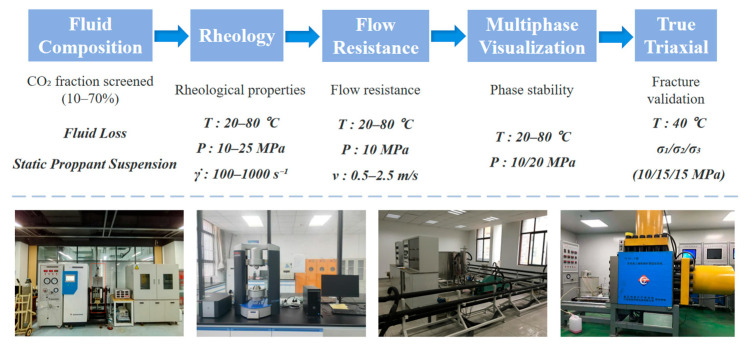
Overview of the experimental workflow and corresponding equipment used in this study under high-temperature and high-pressure conditions.

All experiments were conducted under high-pressure and temperature-controlled conditions. Filtration tests were performed using a CO_2_ high-temperature and high-pressure displacement apparatus. Suspension stability and multiphase visualization experiments were carried out using a high-temperature and high-pressure visual stirring reactor. Rheological measurements were conducted using a high-temperature and high-pressure rotational rheometer (HAAKE MARS 60, Thermo Scientific, Waltham, MA, USA). Flow resistance experiments were performed using a high-temperature and high-pressure tubular flow apparatus. Triaxial fracturing experiments were conducted using a true triaxial fracturing system. These experimental setups were arranged to follow the overall experimental workflow of the study, including fluid composition screening, rheological characterization, flow resistance evaluation, multiphase visualization, and true triaxial fracturing validation, as schematically summarized in Figure 2.

### 2.2. Experimental Methods

#### 2.2.1. Fluid Loss and Proppant Carrying Capacity Tests

Fluid loss and proppant carrying capacity tests were conducted to identify a suitable CO_2_–water mixing ratio range for the CO_2_–water polymer hybrid fracturing fluid. For the fluid loss experiments, artificial sandstone cores with a permeability of 6 mD were used. The cores were cylindrical, with dimensions of 50 mm in diameter and 25 mm in length.

The polymer-based aqueous phase consisted of a 0.35 wt% polymer solution, which falls within the typical concentration range (0.30–0.40 wt%) used in field applications and was selected as a representative value. The CO_2_ volume fraction in the CO_2_–water polymer hybrid fracturing fluid was set to 10%, 30%, 40%, 50%, and 70%. Field applications commonly employ CO_2_ fractions in the range of 30–50%; therefore, this study retained the field-relevant range and further extended it to lower and higher values to evaluate the influence of CO_2_ content on fluid loss behavior.

All fluid loss experiments were conducted at a temperature of 40 °C. The displacement pressure differential was maintained at 3.5 MPa, and the back pressure was fixed at 10 MPa. During each experiment, the cumulative filtrate mass (m) was recorded as a function of time (t). The fluid loss coefficient (C) was calculated using

(1) $$C=0.005\times \frac{m}{A}$$

where A is the cross-sectional area of the core. The fluid loss rate (v_c_) was then calculated using

(2) $$\upsilon_{C}=\frac{C}{\sqrt{t}}$$

The fluid loss coefficient and fluid loss rate were used to compare fluid loss behavior under different CO_2_ volume fractions.

Based on the fluid loss results, CO_2_ volume fractions of 30%, 40%, and 50% were selected for subsequent proppant carrying capacity tests. These experiments were conducted using a high-temperature and high-pressure visual stirring reactor. The system pressure was maintained at 10 MPa, while the temperature varied over six levels (20, 40, 50, 60, 70, and 80 °C). After fluid preparation, the system was maintained under static conditions, and the settling behavior of the proppant bed was recorded as a function of time.

The vertical settling distance (h) over a given time interval (t) was measured, and the proppant settling velocity (v) was calculated using

(3) $$\nu =\frac{h}{t}$$

The settling velocity was used to characterize the proppant carrying capacity of the CO_2_–water polymer hybrid fracturing fluid under different CO_2_ volume fractions and temperature conditions.

#### 2.2.2. Rheological Measurements

Rheological measurements were conducted using a high-temperature and high-pressure rotational rheometer. The experimental pressure ranged from 7 to 25 MPa, and four temperature levels (20, 40, 60, and 80 °C) were investigated.

The CO_2_–water polymer hybrid fracturing fluid consisted of 60% polymer-based aqueous phase and 40% CO_2_ by volume. Rheological tests were performed at shear rates (γ̇) of 100, 170, 200, 500, 700, and 1000 s^−1^. At each shear rate, the fluid was sheared continuously for 10 min to reach steady-state conditions.

During testing, the apparent viscosity was continuously recorded. For each temperature–pressure–shear rate condition, viscosity data from the steady-state region were selected, defined as the interval in which the viscosity variation was ≤1.0 mPa·s. The average viscosity within this interval was taken as the representative viscosity.

#### 2.2.3. Flow Resistance Measurements

Flow resistance measurements were conducted using a high-temperature and high-pressure tubular flow apparatus. The experimental pressure was fixed at 10 MPa, while the temperature was varied over seven levels (20, 30, 40, 50, 60, 70, and 80 °C).

The CO_2_–water polymer hybrid fracturing fluid consisted of 60% polymer-based aqueous phase and 40% CO_2_ by volume. The flow velocity was varied over five levels (0.5, 1.0, 1.5, 2.0, and 2.5 m·s^−1^). The length of the pressure measurement section was 1 m, and the inner diameter of the test tube was 6 mm.

During each experiment, the pressure drop (ΔP) across the measurement section was recorded under steady flow conditions. The Darcy friction factor (f) was calculated according to the Darcy–Weisbach equation:

(4) $$f=\frac{2d}{\rho u^{2}L}\Delta P$$

where d is the inner diameter of the tube, ΔP is the measured pressure drop, ρ is the fluid density, u is the average flow velocity, and L is the length of the test section. The density of the CO_2_–water polymer hybrid fracturing fluid (ρ) was calculated as the weighted average of the densities of the polymer-based aqueous phase and CO_2_ at the corresponding experimental temperature and pressure conditions, with the CO_2_ density obtained using the CoolProp library in Python 3.11.

#### 2.2.4. Multiphase Visualization Experiments

Multiphase visualization experiments were conducted using a high-temperature and high-pressure visual stirring reactor. The temperature was set at four levels (20, 40, 60, and 80 °C), and two pressure levels (10 and 20 MPa) were investigated.

The CO_2_–water polymer hybrid fracturing fluid consisted of the polymer-based aqueous phase and CO_2_ at the designed mixing ratio. During the experiments, a high-resolution camera was used to record images of the hybrid fluid through the optical windows of the visual reactor. Images were captured at three vertical positions within the reactor, corresponding to the upper, middle, and lower regions.

The acquired images were used for qualitative analysis of the multiphase distribution characteristics and macroscopic phase behavior of the CO_2_–water polymer hybrid fracturing fluid under different temperature and pressure conditions.

#### 2.2.5. True Triaxial Fracturing Experiments

True triaxial fracturing experiments were conducted using cubic shale specimens with dimensions of 300 × 300 × 300 mm and a permeability of 0.01 mD. Three principal stresses were independently applied, with magnitudes of σ_1_/σ_2_/σ_3_ = 10/15/15 MPa. The CO_2_–water polymer hybrid fracturing fluid was injected into the shale specimen at a constant flow rate of 60 mL·min^−1^, and the injection pressure was continuously recorded as a function of time during the fracturing process. After completion of the experiment, the fractured shale specimen was retrieved, and the post-fracture morphology was visually examined to characterize fracture development.

## 3. Experimental Results and Discussion

### 3.1. Fluid Loss Behavior of the CO_2_–Water Polymer Hybrid Fracturing Fluid

**Figure 3:**
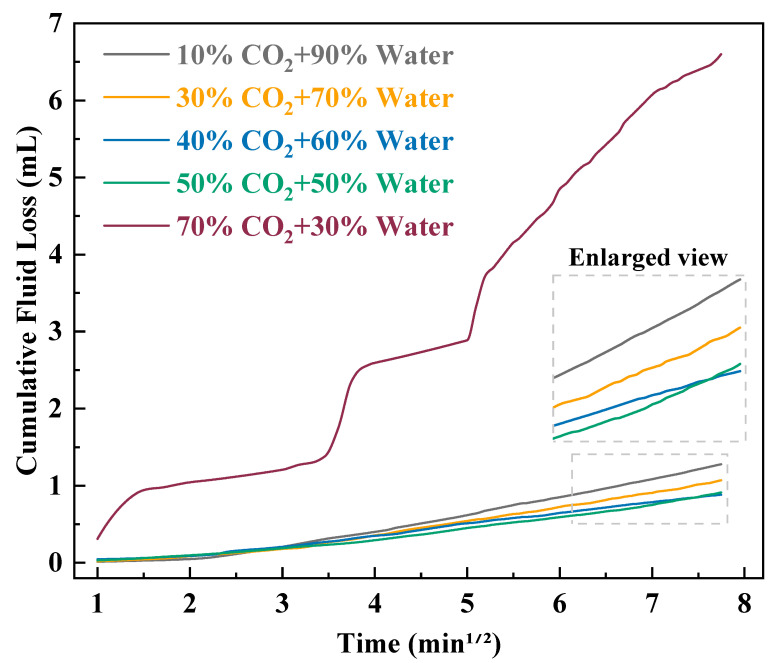
Cumulative fluid loss of the CO_2_–water polymer hybrid fracturing fluid at different CO_2_ volume fractions under 40 °C and a back pressure of 10 MPa.

Under a temperature of 40 °C and a back pressure of 10 MPa, the fluid loss coefficient, fluid loss rate, and cumulative fluid loss of the CO_2_–water polymer hybrid fracturing fluid exhibited pronounced variations with different CO_2_ volume fractions, as summarized in Table 1 and illustrated in Figure 3. As the CO_2_ fraction increased from 10% to 40%, the fluid loss coefficient decreased from 0.2093 to 0.1382, and the fluid loss rate decreased from 4.26 × 10^−4^ to 2.82 × 10^−4^ m·min^−0.5^. Correspondingly, the cumulative fluid loss curves shifted downward, indicating a progressive improvement in fluid loss control. When the CO_2_ fraction was 40% and 50%, the fluid loss coefficients (0.1382 and 0.1380, respectively), and fluid loss rates (2.82 × 10^−4^ and 2.81 × 10^−4^ m·min^−0.5^, respectively), were nearly identical, and the cumulative fluid loss curves almost overlapped, suggesting that the fluid loss behavior of the system became relatively stable within this range. In contrast, when the CO_2_ fraction was further increased to 70%, both the fluid loss coefficient and fluid loss rate increased markedly, reaching 1.0607 and 2.161 × 10^−3^ m·min^−0.5^, respectively. As a result, the cumulative fluid loss became significantly higher than that under other conditions, exhibiting a distinctly different fluid loss behavior compared with low and intermediate CO_2_ fractions.

Based on the experimental results, the influence of CO_2_ volume fraction on the fluid loss behavior of the hybrid fracturing fluid can be characterized by an initial improvement followed by a pronounced deterioration. Within the CO_2_ volume fraction range of 10–40%, increasing the CO_2_ content led to a gradual reduction in the fluid loss coefficient and fluid loss rate, indicating enhanced fluid loss control performance. In the range of 40–50%, the fluid loss parameters remained nearly unchanged, suggesting that the contribution of the polymer-based aqueous phase to fluid loss control had been effectively established and that further adjustment of the mixing ratio had a limited impact on fluid loss behavior. However, at a CO_2_ fraction of 70%, the fluid loss parameters increased by more than one order of magnitude, indicating that excessive CO_2_ content weakened the effective role of the polymer-based aqueous phase in controlling fluid loss, resulting in a significant decline in fluid loss performance.

Based on these consistent trends in fluid loss behavior, and considering both fluid loss control and system stability, CO_2_ volume fractions of 30%, 40%, and 50% were identified as favorable candidates for subsequent proppant carrying capacity and rheological evaluations.

### 3.2. Proppant Carrying Capacity of the CO_2_–Water Polymer Hybrid Fracturing Fluid

**Figure 4:**
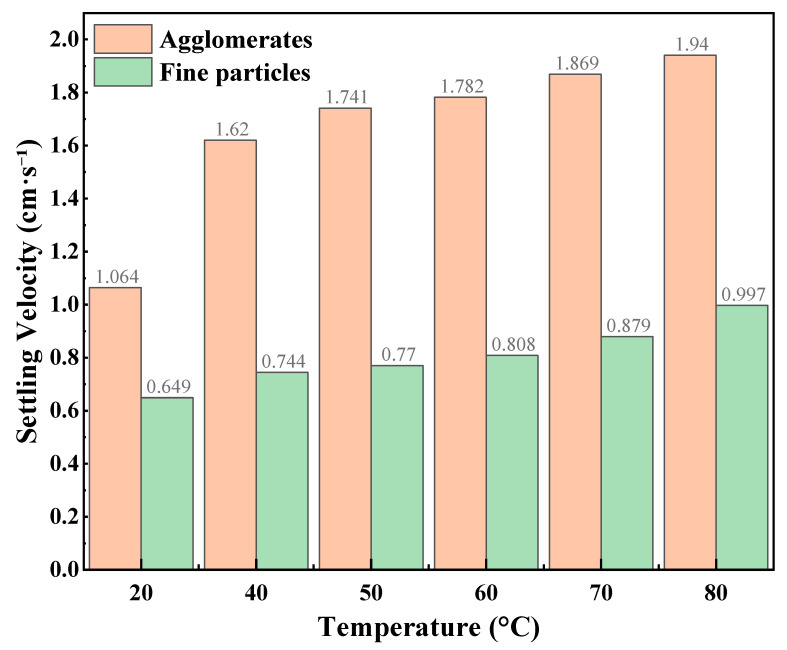
Static proppant settling velocities at different temperatures (30% CO_2_).

**Figure 5:**
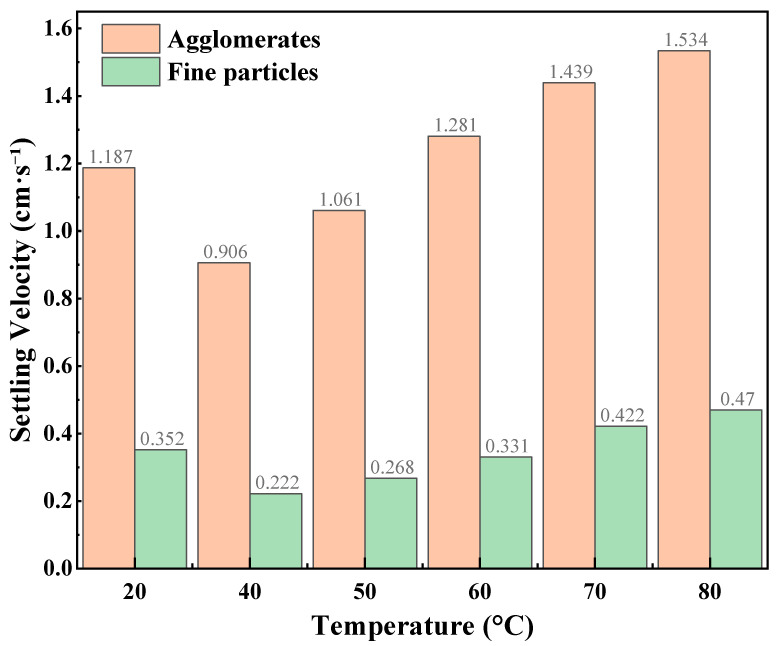
Static proppant settling velocities at different temperatures (40% CO_2_).

**Figure 6:**
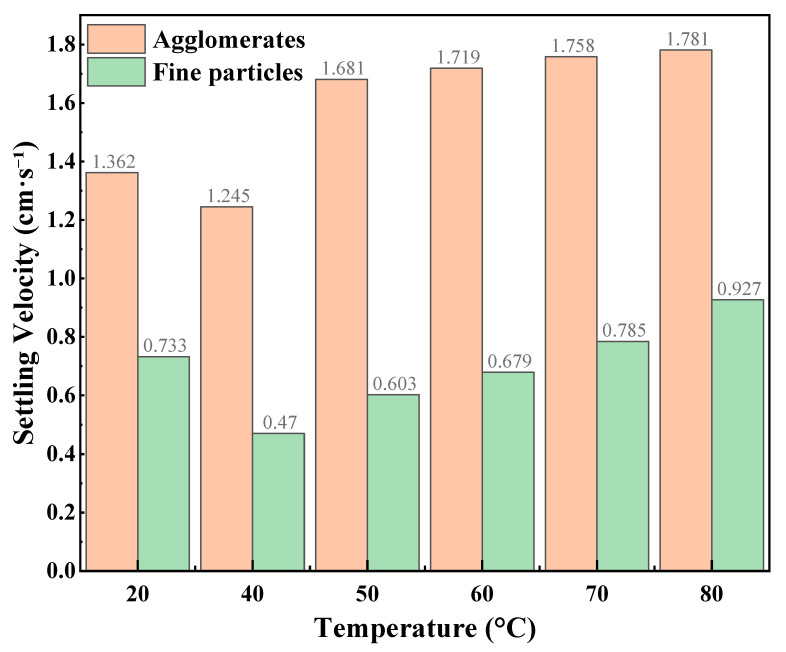
Static proppant settling velocities at different temperatures (50% CO_2_).

The static proppant suspension results of the CO_2_–water polymer hybrid fracturing fluid at different CO_2_ volume fractions are summarized in Table 2 and illustrated in Figure 4, Figure 5 and Figure 6, corresponding to CO_2_ volume fractions of 30%, 40%, and 50%, respectively. Within the investigated temperature range of 20–80 °C, two distinct settling behaviors were consistently observed for all mixing ratios, namely agglomerates and fine particles. For all cases, the settling velocities of agglomerates were higher than those of fine particles, indicating clear differences in the settling characteristics of the two particle types in the mixed-phase system.

At a CO_2_ volume fraction of 30%, the settling velocities of both agglomerates and fine particles increased monotonically with temperature. Specifically, the agglomerate settling velocity increased from 1.064 to 1.94 cm·s^−1^, while the fine-particle settling velocity increased from 0.649 to 0.997 cm·s^−1^, suggesting that the static proppant carrying capacity of the system was relatively sensitive to temperature under this condition. When the CO_2_ volume fraction was 40%, the agglomerate settling velocity slightly decreased at lower temperatures (20–40 °C) and then gradually increased with further temperature elevation. In contrast, the settling velocities of fine particles remained consistently low over the entire temperature range (0.222–0.470 cm·s^−1^) and exhibited relatively weak temperature dependence. For the fluid containing 50% CO_2_, the agglomerate settling velocities remained comparatively high and increased with temperature, while the fine-particle settling velocities also showed a pronounced increasing trend as temperature increased.

At the same temperature, pronounced differences in settling velocities were observed among different CO_2_ volume fractions, particularly for fine particles, indicating that the static suspension behavior of the hybrid fracturing fluid was strongly dependent on the mixing ratio. As lower settling velocities generally correspond to stronger static proppant carrying capacity, the settling behavior of fine particles can be regarded as a sensitive indicator of suspension performance. A comparative analysis shows that under the 40% CO_2_ condition, fine-particle settling velocities were consistently lower and exhibited reduced temperature sensitivity compared with those observed at 30% and 50% CO_2_. This behavior suggests a more stable contribution of the polymer-based aqueous phase in restraining particle settling at this mixing ratio.

Combined with the preliminary screening based on fluid-loss behavior, the static proppant-suspension results further support the selection of a hybrid fracturing fluid composed of 60% polymer-based aqueous phase and 40% CO_2_. This mixing ratio was therefore adopted for subsequent rheological measurements, flow resistance evaluation, multiphase visualization experiments, and true triaxial fracturing tests.

### 3.3. Rheological Behavior

**Figure 7:**
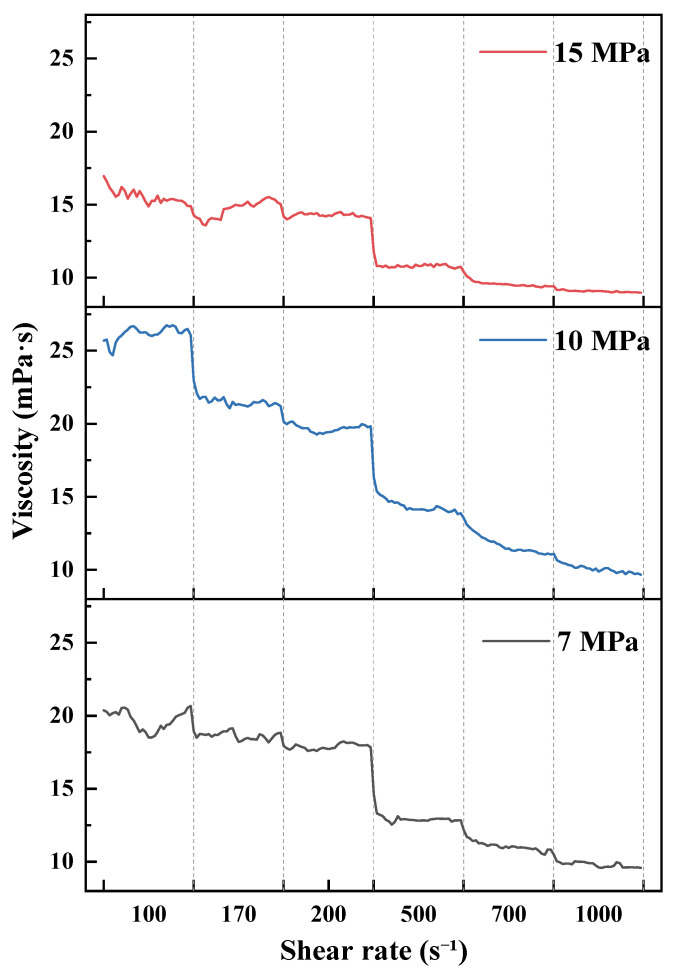
Viscosity evolution at 20 °C under stepwise shear and different pressures.

**Figure 8:**
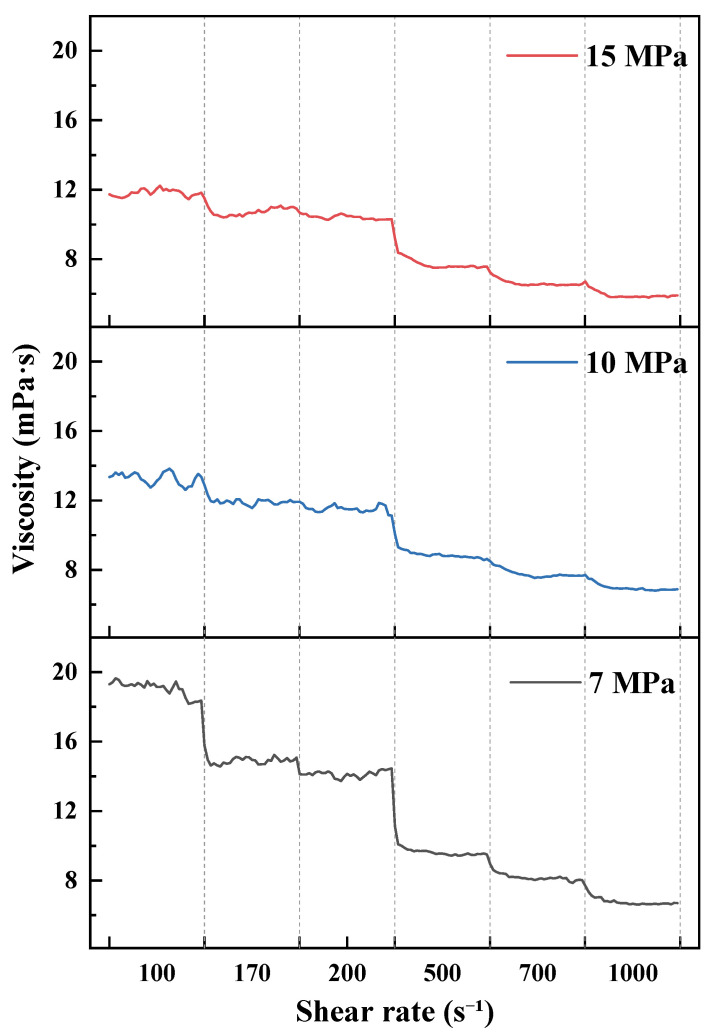
Viscosity evolution at 40 °C under stepwise shear and different pressures.

**Figure 9:**
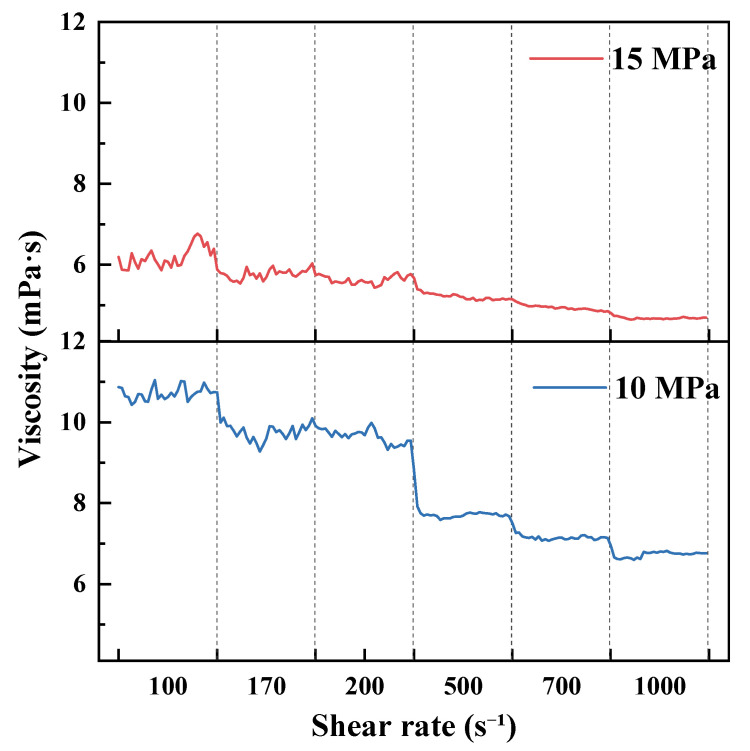
Viscosity evolution at 60 °C under stepwise shear and different pressures.

**Figure 10:**
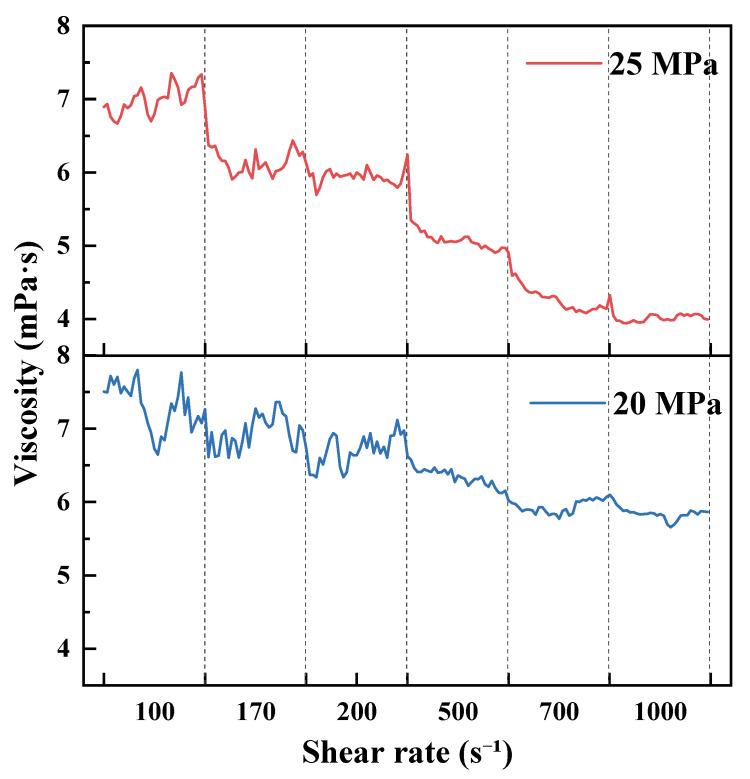
Viscosity evolution at 80 °C under stepwise shear and different pressures.

As shown in Figure 7, Figure 8, Figure 9 and Figure 10 and summarized by the steady-state viscosity values in Table 3, the CO_2_–water polymer hybrid fracturing fluid composed of 60% polymer-based aqueous phase and 40% CO_2_ exhibits pronounced shear-thinning behavior under all investigated temperature and pressure conditions. As the shear rate increases stepwise from 100 to 1000 s^−1^, the apparent viscosity decreases continuously, with rapid stabilization observed after each shear-rate transition.

Taking the condition at 20 °C as an example (Figure 7 and Table 3), the apparent viscosity at 7 MPa decreases from 19.70 mPa·s at 100 s^−1^ to 9.82 mPa·s at 1000 s^−1^. At 10 MPa, the viscosity decreases from 26.17 to 10.10 mPa·s, while at 15 MPa it decreases from 15.56 to 9.08 mPa·s over the same shear-rate range. Similar shear-thinning behavior is observed at elevated temperatures of 40, 60, and 80 °C (Figure 8, Figure 9 and Figure 10), indicating that the hybrid fracturing fluid maintains stable shear-responsive characteristics under high-temperature and high-pressure conditions.

Temperature exerts a significant influence on the viscosity level of the CO_2_–water polymer hybrid fracturing fluid. At comparable shear rates and pressures, the apparent viscosity decreases systematically with increasing temperature. For example, at a shear rate of 100 s^−1^ and a pressure of 15 MPa, the viscosity decreases from 15.56 mPa·s at 20 °C to 11.81 mPa·s at 40 °C, and further to 6.21 mPa·s at 60 °C (Table 3), reflecting reduced flow resistance at elevated temperatures.

In contrast, the effect of pressure on apparent viscosity does not follow a strictly monotonic trend and exhibits clear temperature dependence. At 20 °C, the viscosity at 10 MPa is higher than that at 7 and 15 MPa, whereas at 40 °C, the viscosity at 7 MPa exceeds that at higher pressures (Figure 7 and Figure 8). This behavior indicates a coupled influence of temperature and pressure on the rheological response of the hybrid fracturing fluid, which is associated with pressure-dependent variations in the CO_2_ phase and its interaction with the polymer-based aqueous phase.

Overall, the rheological results demonstrate that the CO_2_–water polymer hybrid fracturing fluid exhibits stable shear-thinning behavior over a wide range of temperatures, pressures, and shear rates. Temperature and pressure primarily modulate the viscosity level without altering the fundamental shear-responsive characteristics of the system. These rheological features are favorable for maintaining good pumpability under high-shear conditions while retaining sufficient viscosity under lower-shear conditions, supporting the applicability of the hybrid fracturing fluid in high-temperature and high-pressure reservoir environments.

### 3.4. Flow Resistance Characteristics

**Figure 11:**
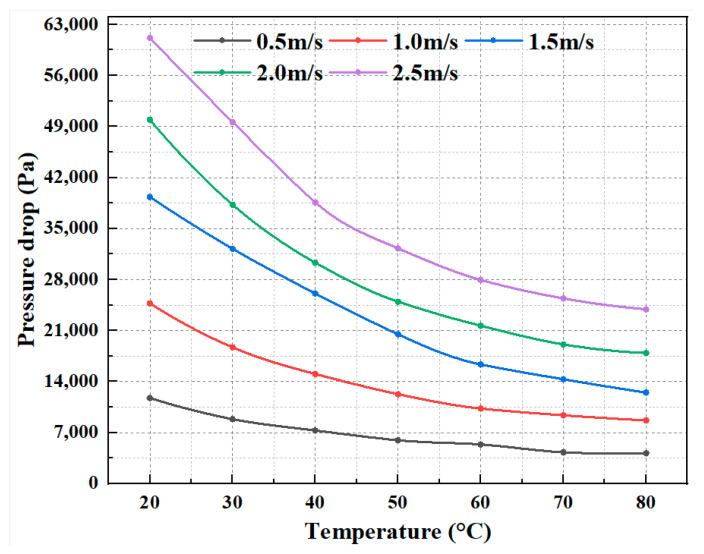
Variation in pressure drop with temperature at different flow velocities for the CO_2_–water polymer hybrid fracturing fluid.

**Figure 12:**
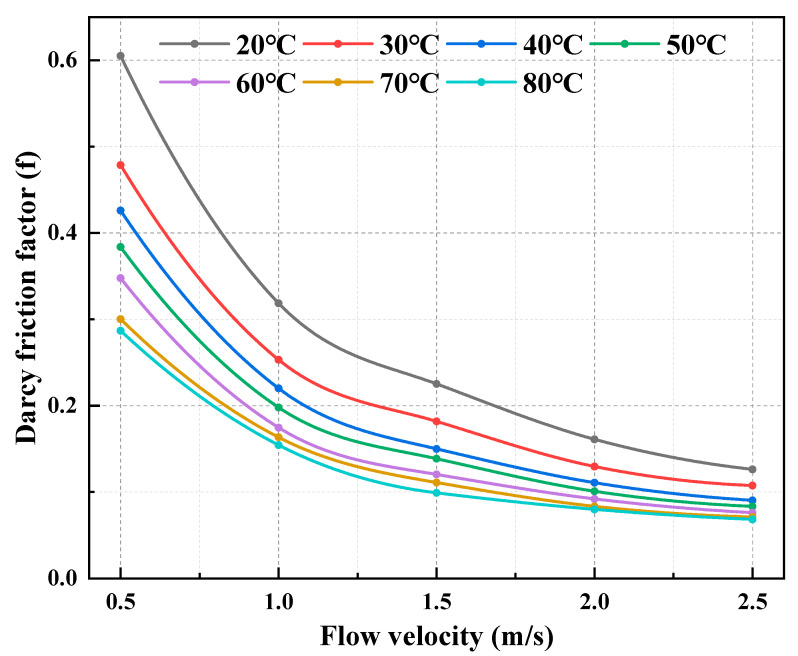
Dependence of Darcy friction factor on flow velocity at different temperatures for the CO_2_–water polymer hybrid fracturing fluid.

**Figure 13:**
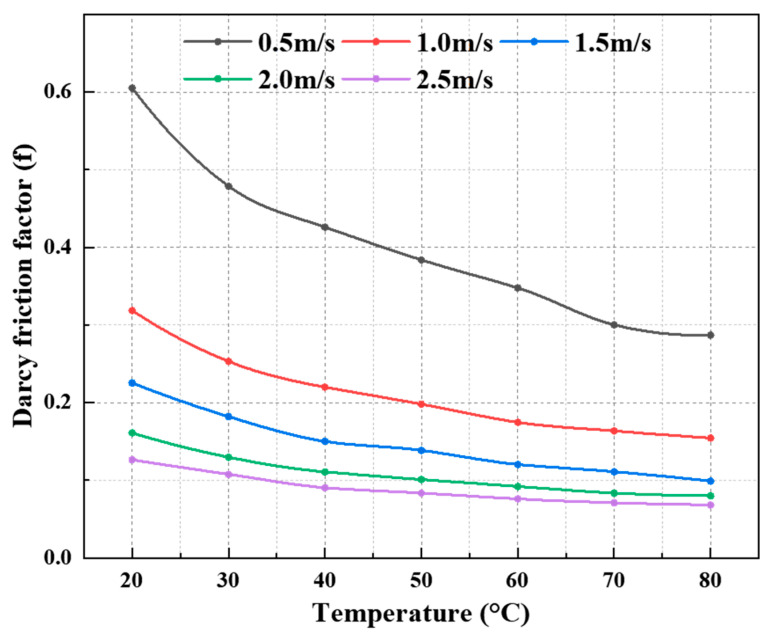
Variation in Darcy friction factor with temperature at different flow velocities for the CO_2_–water polymer hybrid fracturing fluid.

The flow resistance behavior of the CO_2_–water polymer hybrid fracturing fluid was investigated over a temperature range of 20–80 °C and flow velocities from 0.5 to 2.5 m·s^−1^. The measured pressure drop, Reynolds number, and calculated Darcy friction factor are summarized in Table 4, and the corresponding variations are illustrated in Figure 11, Figure 12 and Figure 13.

At all investigated temperatures, the pressure drop increases monotonically with increasing flow velocity, whereas the Darcy friction factor decreases continuously with increasing Reynolds number. Taking 20 °C as an example, when the flow velocity increases from 0.5 to 2.5 m·s^−1^, the Reynolds number increases from 214.1 to 2085.0, accompanied by a decrease in the friction factor from 0.6050 to 0.1261. Similar trends are observed at elevated temperatures. For instance, at 60 °C, the friction factor decreases from 0.3646 (Re = 535.1) to 0.0759 (Re = 4476.8), while at 80 °C it decreases from 0.2968 (Re = 331.8) to 0.0682 (Re = 3800.5). These results indicate that the CO_2_–water polymer hybrid fracturing fluid exhibits progressively reduced flow resistance with increasing flow velocity within the investigated Reynolds number range.

Temperature has a pronounced effect on the overall magnitude of flow resistance. At a given flow velocity, both the pressure drop and Darcy friction factor decrease systematically with increasing temperature, while the Reynolds number increases accordingly. For example, at a flow velocity of 1.0 m·s^−1^, the friction factor decreases from 0.3186 at 20 °C to 0.1745 at 60 °C, and further to 0.1543 at 80 °C. This temperature-dependent trend is consistently observed across the entire velocity range, as shown in Table 4 and Figure 11, Figure 12 and Figure 13.

The observed flow resistance characteristics are primarily governed by variations in the apparent viscosity of the CO_2_–water polymer hybrid fracturing fluid. As demonstrated by the rheological results, the system exhibits pronounced shear-thinning behavior; increasing flow velocity leads to a reduction in effective viscosity and, consequently, a decrease in the friction factor. In addition, increasing the temperature further reduces the viscosity of the polymer-based aqueous continuous phase, resulting in lower flow resistance. Within the investigated temperature and Reynolds number ranges, the combined effects of shear and temperature dominate the flow resistance behavior of the hybrid fracturing fluid, while the presence of CO_2_ contributes to modifying the multiphase flow structure without altering the overall decreasing trend of friction with increasing flow velocity.

### 3.5. Phase Visualization and Evolution of the CO_2_–Water Polymer Hybrid Fracturing Fluid

#### 3.5.1. Initial Phase Appearance of the CO_2_–Water Polymer Hybrid Fracturing Fluid After Mixing

The initial phase appearance of the CO_2_–water polymer hybrid fracturing fluid after mixing was visualized at temperatures of 20, 40, 60, and 80 °C and pressures of 10 and 20 MPa. For each condition, images were captured at the upper, middle, and lower positions of the visual cell to characterize the macroscopic vertical distribution of the system. Overall, within the temperature range of 20–60 °C, the hybrid fracturing fluid exhibited a relatively uniform milky appearance immediately after mixing under both 10 and 20 MPa. No obvious phase stratification or macroscopic separation was observed among the upper, middle, and lower regions, indicating good initial macroscopic homogeneity of the system within this temperature–pressure range. Corresponding images are provided in the Supplementary Materials.

**Figure 14:**
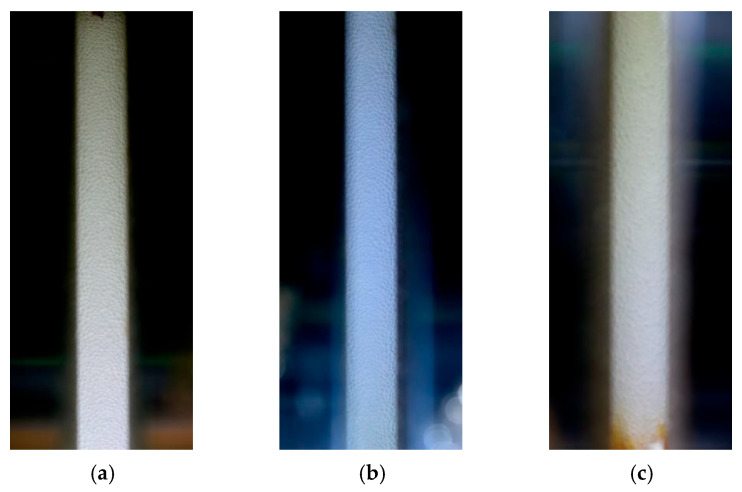
Phase appearance at 80 °C and 10 MPa: (**a**) upper; (**b**) middle; (**c**) lower.

**Figure 15:**
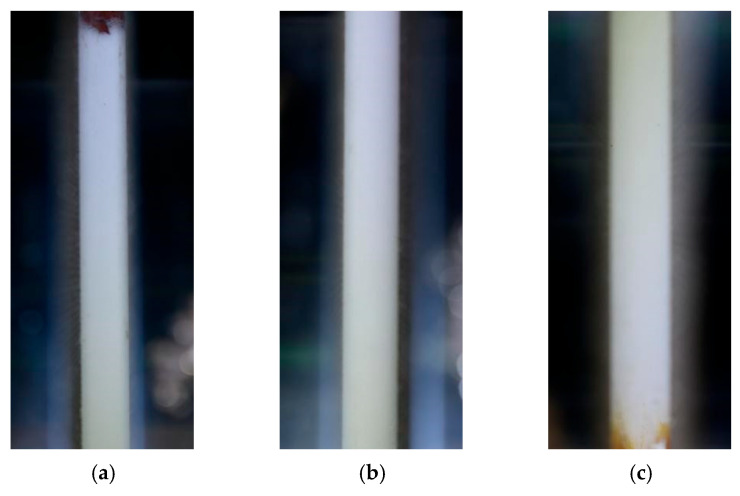
Phase appearance at 80 °C and 20 MPa: (**a**) upper; (**b**) middle; (**c**) lower.

When the temperature increased to 80 °C, the initial phase appearance of the system showed a pronounced dependence on pressure. Under the condition of 80 °C and 10 MPa, clearly distinguishable dispersed bubble-like structures were observed throughout the visual cell, including the upper, middle, and lower regions (Figure 14), indicating a significant change in macroscopic phase appearance under high-temperature and relatively low-pressure conditions. In contrast, under 80 °C and 20 MPa, the hybrid fracturing fluid maintained a relatively uniform appearance across different vertical positions, and no obvious macroscopic phase separation was observed (Figure 15), demonstrating that increased pressure exerts a stabilizing effect on the initial phase appearance of the system.

These observations indicate that the initial phase appearance of the CO_2_–water polymer hybrid fracturing fluid after mixing is strongly influenced by the combined effects of temperature and pressure. Under lower temperatures or higher pressures, the system tends to form a uniform mixed-phase structure, whereas under high-temperature and relatively low-pressure conditions, changes in the phase state and distribution of CO_2_ lead to the emergence of a bubble-like dispersed structure at the macroscopic scale. This behavior provides a critical basis for the subsequent analysis of phase evolution and stability.

#### 3.5.2. Phase Stability Evolution of the Hybrid Fracturing Fluid Under Static Conditions

**Figure 16:**
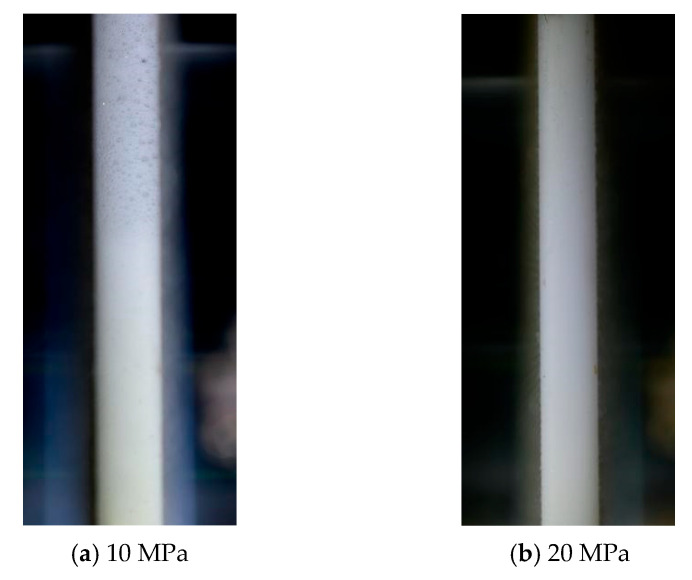
Phase appearance of the CO_2_–water polymer hybrid fracturing fluid at the upper region after standing for 10 min at 60 °C under 10 and 20 MPa.

**Figure 17:**
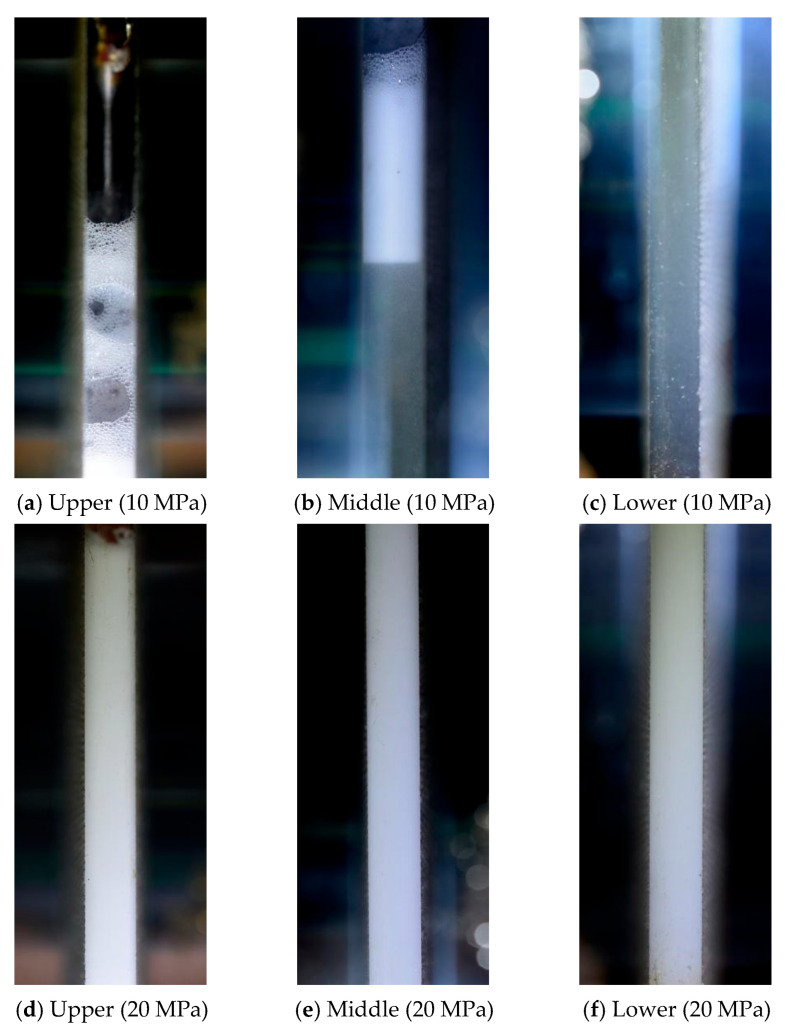
Phase appearance of the CO_2_–water polymer hybrid fracturing fluid at the upper, middle, and lower regions after standing for 5 min at 80 °C under 10 and 20 MPa.

After high-temperature and high-pressure mixing, the CO_2_–water polymer hybrid fracturing fluid was allowed to stand under quiescent conditions to evaluate the evolution of its phase stability in the absence of shear. Figure 16 presents the phase appearance of the hybrid fracturing fluid in the upper region of the visual cell after standing for 10 min at 60 °C under pressures of 10 and 20 MPa, while Figure 17 shows the phase distribution in the upper, middle, and lower regions of the visual cell after standing for 5 min at 80 °C under the same pressure levels.

Overall, under moderate temperature or elevated pressure conditions, the hybrid fracturing fluid was able to maintain a dispersed-phase-dominated macroscopic structure over short time scales during static standing. At 60 °C and 10 MPa (Figure 16a), the fluid in the upper region retained an overall milky appearance after standing for 10 min; however, loosely distributed bubbles were observed in the upper portion, whereas the lower portion remained dominated by a continuous milky dispersed phase, and no distinct gas–liquid interface was formed. This indicates that under this temperature–pressure condition, early-stage gas exsolution and local bubble aggregation had initiated, although the system was still predominantly characterized by a dispersed structure. In contrast, at 60 °C and 20 MPa (Figure 16b), the upper region maintained a relatively uniform milky appearance after standing for 10 min, with no obvious bubble accumulation or phase separation observed, suggesting enhanced short-term phase stability under elevated pressure.

When the temperature was further increased to 80 °C, the differences in phase stability under different pressure conditions became markedly more pronounced (Figure 17). At 80 °C and 10 MPa, a clear vertical differentiation of phase appearance was observed after standing for 5 min. The upper region was dominated by bubble-rich structures, indicating significant gas accumulation. In the middle region, the upper portion was primarily characterized by bubble-like structures, while the lower portion gradually transitioned into a relatively clear aqueous continuous phase. Meanwhile, the lower region was almost entirely occupied by the polymer-based aqueous phase, with no obvious bubbles or milky dispersed structures observed. This vertical distribution, characterized by a gradual transition from gas-rich regions at the top to liquid-dominated regions at the bottom, demonstrates that under high-temperature and relatively low-pressure conditions, the CO_2_–water polymer hybrid fracturing fluid system undergoes rapid gas–liquid separation accompanied by gravity-driven phase rearrangement during static standing.

By comparison, at 80 °C and 20 MPa, the fluid in the upper, middle, and lower regions of the visual cell generally maintained a continuous milky appearance after standing for 5 min, with only minor local brightness variations and no distinct gas–liquid interfaces or obvious stratification observed. This indicates that under high-temperature conditions, increasing pressure can effectively suppress rapid CO_2_ exsolution and aggregation, thereby delaying the destabilization of the hybrid phase structure during static conditions.

Overall, the phase stability of the CO_2_–water polymer hybrid fracturing fluid under static conditions is predominantly governed by the coupled effects of temperature and pressure. At moderate temperatures or elevated pressures, the system can maintain a dispersed-phase-dominated structure over short time scales. In contrast, under high-temperature and relatively low-pressure conditions, the system tends to evolve from an initially milky dispersed state toward bubble-rich structures and ultimately to distinct gas–liquid separation, accompanied by pronounced spatial heterogeneity.

#### 3.5.3. Discussion and Analysis of Visualized Phase Behavior

Based on the visualized results obtained under different temperature, pressure, and static standing conditions, the CO_2_–water polymer hybrid fracturing fluid exhibits a pronounced dependence of its macroscopic phase behavior on operating conditions and time. Although the fluid generally presents a milky and visually homogeneous appearance immediately after mixing, this state is not necessarily stable during subsequent static standing, and its evolution varies significantly with temperature and pressure.

The milky appearance observed immediately after mixing is associated with a highly dispersed distribution of CO_2_ within the polymer-based aqueous phase. Under stirring, CO_2_ is present in the form of dispersed gas bubbles or fine-scale dispersed structures within the continuous polymer-based aqueous phase, leading to an apparently uniform macroscopic appearance. The presence of the polymer-based aqueous phase contributes to maintaining this dispersed structure and retarding the aggregation and migration of the dispersed CO_2_ domains over short time scales.

As temperature increases and/or pressure decreases, the dispersed structure becomes progressively less stable under static conditions. Under intermediate conditions, such as 60 °C and 10 MPa, the appearance of locally distributed bubbles indicates the onset of structural weakening, although the system remains predominantly characterized by a dispersed-phase-dominated structure. When the temperature is further increased under relatively low pressure, the phase evolution of the system is accelerated, resulting in the formation of bubble-rich regions and pronounced vertical differentiation, reflecting gravity-driven redistribution during static standing.

In contrast, increasing pressure under the same high-temperature conditions effectively delays the destabilization of the dispersed structure. Even at elevated temperatures, the fluid can maintain a relatively uniform milky appearance over short static periods when subjected to higher pressures. This observation highlights the important role of pressure in moderating the phase behavior and short-term stability of the CO_2_–water polymer hybrid fracturing fluid.

Overall, the visualized phase behavior indicates that the milky appearance of the CO_2_–water polymer hybrid fracturing fluid corresponds to a kinetically sustained dispersed structure rather than a true single-phase state. The evolution of this structure under static conditions is governed by the combined influence of temperature, pressure, and gravity, which collectively control the redistribution of the dispersed CO_2_ within the hybrid system.

### 3.6. True Triaxial Fracturing Behavior of the CO_2_–Water Polymer Hybrid Fracturing Fluid

**Figure 18:**
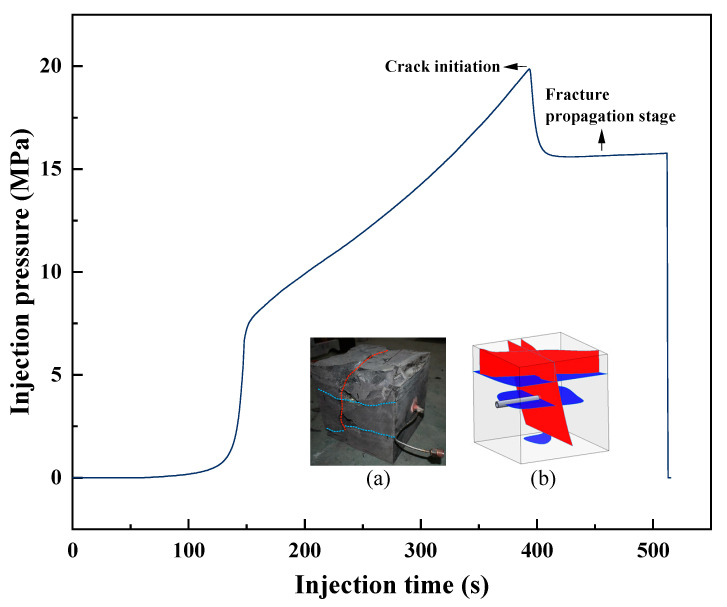
Injection pressure evolution during true triaxial fracturing of the shale sample, together with (**a**) the post-fracture specimen and (**b**) the corresponding schematic illustration of fracture geometry. The blue lines represent bedding-parallel fractures, while the red lines represent bedding-crossing fractures.

Figure 18 presents the injection pressure evolution during true triaxial fracturing of the shale specimen using the CO_2_–water polymer hybrid fracturing fluid, together with the post-fracture specimen and a schematic illustration of the resulting fracture geometry. The shale specimen had dimensions of 300 × 300 × 300 mm and a permeability of approximately 0.01 mD. The applied true triaxial stresses were σ_1_/σ_2_/σ_3_ = 10/15/15 MPa, and the fracturing fluid was injected along the minimum principal stress direction. Prior to injection, the hybrid fracturing fluid was prepared at 40 °C and 10 MPa and subsequently injected at a constant flow rate of 60 mL·min^−1^.

The injection pressure curve exhibits a typical evolution process commonly observed in hydraulic fracturing experiments, including pressure buildup, crack initiation, pressure drop, and a subsequent fracture propagation stage. During the initial injection period, the pressure gradually increased with continuous fluid injection, reflecting the pressurization of the borehole and the surrounding shale matrix. When the injection pressure reached approximately 19.85 MPa, a distinct pressure peak was observed, indicating the initiation of fracturing.

Following crack initiation, the injection pressure decreased rapidly and then stabilized at a relatively constant level of approximately 15.5 MPa during the fracture propagation stage. The pressure difference between the initiation peak and the propagation stage was about 4.35 MPa. This pressure response is consistent with the sudden increase in flow area associated with fracture opening, followed by sustained fracture extension under relatively stable hydraulic conditions.

The post-fracture specimen and the corresponding schematic illustration indicate that fracture propagation was influenced by both the applied stress conditions and the shale bedding structure. The fractures propagated preferentially along bedding planes while also extending across bedding interfaces in certain regions, resulting in a fracture geometry characterized by both bedding-parallel opening and cross-bedding penetration. This fracture morphology suggests that bedding planes acted as mechanically weak interfaces, while continued fluid injection enabled fracture growth beyond individual bedding layers.

Overall, the injection pressure evolution and the observed fracture morphology demonstrate that the CO_2_–water polymer hybrid fracturing fluid was capable of inducing fracture initiation and sustaining fracture propagation under true triaxial stress conditions. The consistency between the pressure response and the post-fracture observations supports the reliability of the experimental results and provides experimental evidence for the applicability of the hybrid fracturing fluid under constrained stress conditions.

## 4. Conclusions

This study experimentally investigated a CO_2_–water polymer hybrid fracturing fluid using an AM/AA copolymer as the aqueous-phase component under high-temperature and high-pressure conditions. A series of fluid loss, static proppant carrying, rheological, flow resistance, phase visualization, and true triaxial fracturing experiments were conducted to evaluate its key flow, stability, and fracturing characteristics.

(1) The results show that the performance of the hybrid fracturing fluid is strongly dependent on the CO_2_–water mixing ratio as well as temperature and pressure conditions. Fluid loss experiments indicate that a CO_2_ volume fraction of 30–50% provides effective fluid loss control, while static proppant carrying tests further identify a composition of 60% polymer-based aqueous phase and 40% CO_2_ as a balanced formulation with favorable suspension stability over a wide temperature range.
(2) Rheological measurements demonstrate that the hybrid fracturing fluid exhibits stable shear-thinning behavior under temperatures of 20–80 °C and pressures of 7–25 MPa, with continuous and reproducible viscosity responses over a wide shear-rate range. Flow resistance tests show predictable frictional behavior without abnormal fluctuations, and the friction factor decreases systematically with increasing flow velocity and temperature, indicating favorable pumpability under the investigated conditions.
(3) Phase visualization experiments reveal that the hybrid fracturing fluid forms a dispersed mixed-phase structure immediately after mixing, with its short-term stability governed by the coupled effects of temperature and pressure. Elevated temperatures combined with relatively low pressures accelerate phase destabilization and gas–liquid separation under static conditions, whereas increasing pressure effectively suppresses CO_2_ exsolution and stratification.
(4) True triaxial fracturing experiments confirm that the CO_2_–water polymer hybrid fracturing fluid is capable of achieving clear fracture initiation and sustained fracture propagation under constrained stress conditions, with fracture development strongly influenced by shale bedding structures.

Overall, the experimental results demonstrate that the AM/AA copolymer can serve as an effective aqueous-phase component in CO_2_–water polymer hybrid fracturing fluids within the investigated conditions. Without relying on specialized CO_2_ thickeners, the polymer-based aqueous phase provides integrated fluid loss control, proppant carrying capability, stable rheological behavior, and predictable flow resistance, supporting the technical feasibility of this hybrid fracturing fluid concept for high-temperature reservoir applications.

## Figures and Tables

**Table 1:** Fluid loss coefficient and fluid loss rate of the CO_2_–water polymer hybrid fracturing fluid at different CO_2_ volume fractions.

CO_2_ Volume Fraction (%)	Fluid Loss Coefficient (mL·min^−0.5^)	Fluid Loss Rate (m·min^−0.5^)
10%	0.2093	0.000426
30%	0.1710	0.000348
40%	0.1382	0.000282
50%	0.1380	0.000281
70%	1.0607	0.002161

**Table 2:** Proppant settling velocity of the CO_2_–water polymer hybrid fracturing fluid at different CO_2_ volume fractions.

CO_2_ Volume Fraction (%)	Settling Type	Settling Velocity (cm·s^−1^)					
		20	40	50	60	70	80
30%	Agglomerates	1.064	1.62	1.741	1.782	1.869	1.94
	Fine particles	0.649	0.744	0.77	0.808	0.879	0.997
40%	Agglomerates	1.187	0.906	1.061	1.281	1.439	1.534
	Fine particles	0.352	0.222	0.268	0.331	0.422	0.47
50%	Agglomerates	1.362	1.245	1.681	1.719	1.758	1.781
	Fine particles	0.733	0.47	0.603	0.679	0.785	0.927

**Table 3:** Apparent viscosity of the CO_2_–water polymer hybrid fracturing fluid under different temperatures, pressures, and shear rates.

Temperature (°C)	Pressure (MPa)	Apparent Viscosity at Different Shear Rates (mPa·s)					
		100 s^−1^	170 s^−1^	200 s^−1^	500 s^−1^	700 s^−1^	1000 s^−1^
20	7	19.70	18.63	17.88	12.96	11.09	9.82
	10	26.17	21.52	19.70	14.40	11.76	10.10
	15	15.56	14.70	14.28	10.82	9.58	9.08
40	7	19.06	14.92	14.13	9.66	8.18	6.79
	10	13.28	11.94	11.52	8.90	7.79	7.00
	15	11.81	10.76	10.41	7.75	6.62	5.95
60	10	10.71	9.79	9.66	7.75	7.16	6.73
	15	6.21	5.78	5.63	5.23	4.94	4.68
80	20	7.30	6.96	6.70	6.34	5.93	5.84
	25	6.99	6.16	5.94	5.11	4.29	4.02

**Table 4:** Pressure drop, Reynolds number, and Darcy friction factor of the CO_2_–water polymer hybrid fracturing fluid at different temperatures and flow velocities.

Temperature (°C)	Flow Velocity (m/s)	Pressure Drop (Pa)	Reynolds Number (Re)	Darcy Friction Factor (f)
20	0.5	11,736.26	214.1	0.6050
	1	24,722.68	570.6	0.3186
	1.5	39,307.69	1012.4	0.2252
	2	49,932.67	1520.7	0.1609
	2.5	61,148.21	2085	0.1261
30	0.5	8832.52	265.1	0.4785
	1	18,681.01	680.4	0.2530
	1.5	32,215.62	1181.1	0.1939
	2	38,258.02	1746.7	0.1295
	2.5	49,606.10	2366.1	0.1075
40	0.5	7281.74	353.4	0.4259
	1	15,040.05	881.3	0.2199
	1.5	26,073.19	1541.5	0.1694
	2	30,321.07	2260.9	0.1108
	2.5	38,594.01	3018.2	0.0903
50	0.5	5937.17	459.3	0.3836
	1	12,254.15	1094.5	0.1979
	1.5	20,489.38	1930.5	0.1471
	2	24,955.63	2814.6	0.1008
	2.5	32,267.29	3677.9	0.0834
60	0.5	5368.90	535.1	0.3646
	1	10,279.15	1335.8	0.1745
	1.5	16,331.91	2281.1	0.1232
	2	21,653.56	3334.7	0.0919
	2.5	27,950.08	4476.8	0.0759
70	0.5	4297.41	503.3	0.3001
	1	9357.14	1316.5	0.1634
	1.5	14,306.57	2310.5	0.1110
	2	19,086.24	3443.8	0.0833
	2.5	25,403.05	4693.3	0.0710
80	0.5	4161.52	331.8	0.2968
	1	8656.42	948.2	0.1543
	1.5	12,480.14	1752.7	0.0989
	2	17,913.76	2710.3	0.0798
	2.5	23,902.83	3800.5	0.0682

## Data Availability

The original contributions presented in this study are included in the article/Supplementary Material. Further inquiries can be directed to the corresponding author.

## Supplementary Materials

The following supporting information can be downloaded at: https://www.mdpi.com/article/10.3390/polym18030418/s1, Figure S1: Phase appearance of the CO_2_–water polymer hybrid fracturing fluid at 20 °C and 10 MPa; Figure S2: Phase appearance at 20 °C and 20 MPa; Figure S3: Phase appearance at 40 °C and 10 MPa; Figure S4: Phase appearance at 40 °C and 20 MPa; Figure S5: Phase appearance at 60 °C and 10 MPa; Figure S6: Phase appearance at 60 °C and 20 MPa.
