# Supplementary material for: Performance of a High-Molecular-Weight AM/AA Copolymer in a CO2–Water Polymer Hybrid Fracturing Fluid Under High-Temperature and High-Pressure Conditions
Source: Polymers (Basel). 2026 Feb 5;18(3):418. doi: 10.3390/polym18030418 (PMC12899513; doi:10.3390/polym18030418)
Supplement: Supplementary file 1 [file polymers-18-00418-s001.zip › polymers-4116002-supplementary.pdf]

Supplementary Materials

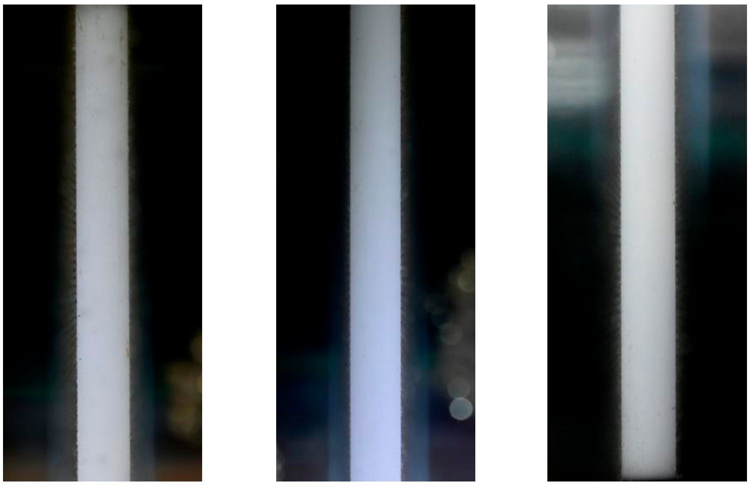

(a) Upper (b) Middle (c) Lower

Figure S1. Phase appearance of the CO_2–water polymer hybrid fracturing fluid at 20 °C and 10 MPa.

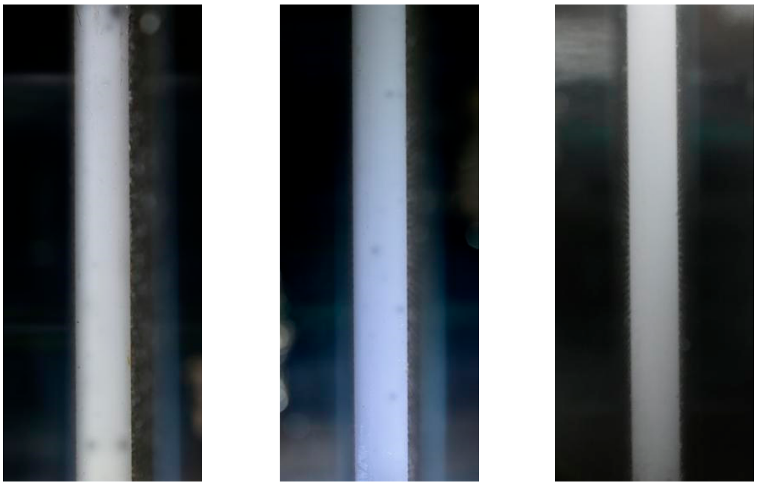

(a) Upper (b) Middle (c) Lower

Figure S2. Phase appearance at 20 °C and 20 MPa.

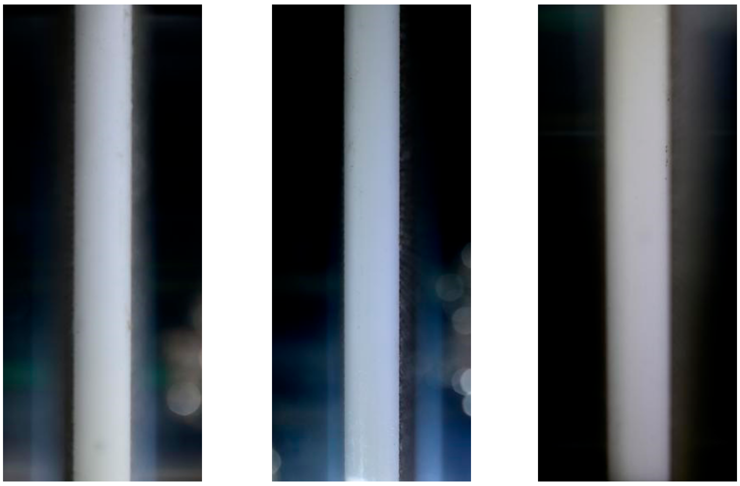

(a) Upper (b) Middle (c) Lower

Figure S3. Phase appearance at 40 °C and 10 MPa.

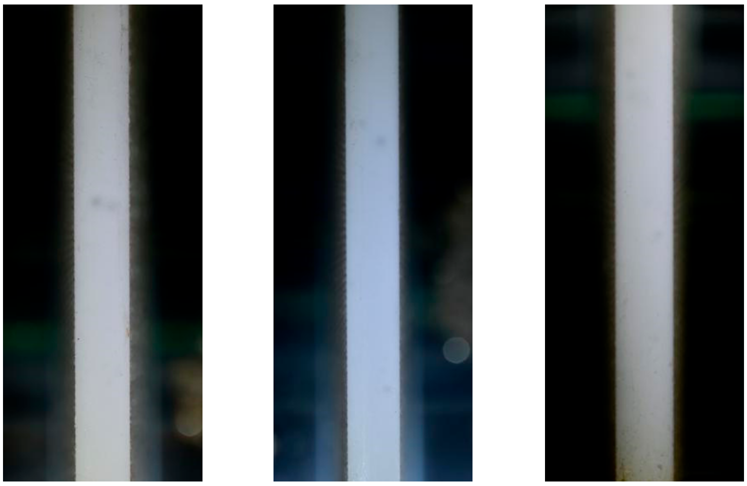

(a) Upper (b) Middle (c) Lower

Figure S4. Phase appearance at 40 °C and 20 MPa.

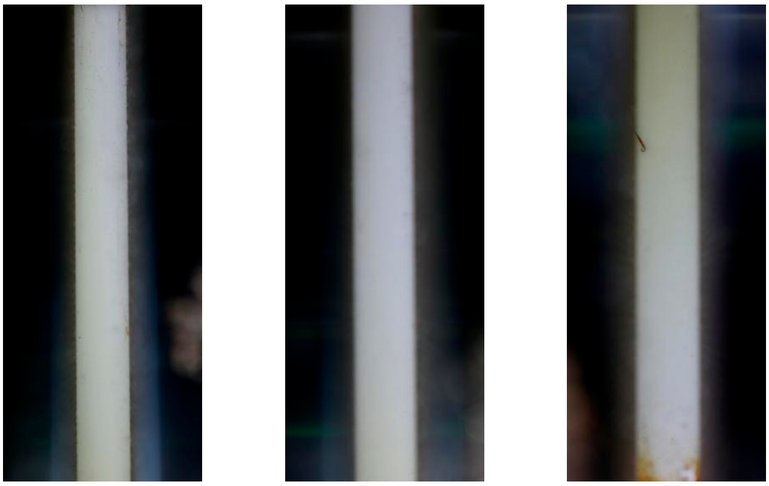

(a) Upper (b) Middle (c) Lower

Figure S5. Phase appearance at 60 °C and 10 MPa.

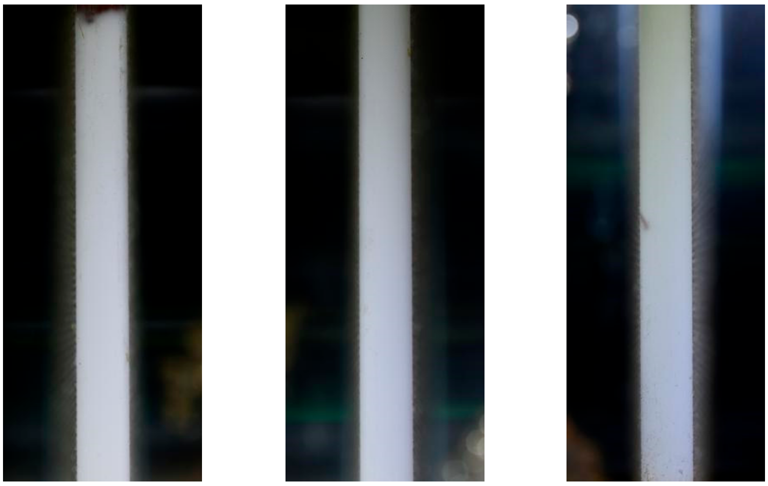

(a) Upper (b) Middle (c) Lower

Figure S6. Phase appearance at 60 °C and 20 MPa.
